# Malignant Glaucoma: A Review of the Modern Literature

**DOI:** 10.1155/2012/852659

**Published:** 2012-03-27

**Authors:** H. Shahid, J. F. Salmon

**Affiliations:** Oxford Eye Hospital, John Radcliffe Hospital, Headington, Oxford OX3 9DU, UK

## Abstract

Malignant glaucoma is a rare form of glaucoma that typically follows surgery in patients with primary angle closure and primary angle-closure glaucoma. In this paper, the clinical features, classification, pathogenesis, and principles of management are discussed. Despite a high prevalence of primary angle closure glaucoma in South-East Asia, the vast majority of cases of malignant glaucoma are reported in White populations. This may reflect differing mechanisms of angle closure in White and Asian patients, which somehow reduces the likelihood of an aberrant relationship developing between the lens, ciliary body, anterior hyaloid, and vitreous structures within the eye. Although the exact underlying pathogenic mechanism remains unclear, the prognosis is good with modern medical, laser, and surgical treatment modalities.

## 1. Introduction

The term “malignant glaucoma” was coined by von Graefe in 1869 to describe an aggressive form of postoperative glaucoma that was resistant to treatment and resulted in blindness [1]. It is alternatively known by names relating to the proposed pathogenic mechanisms of this condition, such as ciliary block glaucoma, aqueous misdirection syndrome, and direct lens-block glaucoma [2–4]. It should be emphasised to patients with malignant glaucoma that the term does not indicate a neoplastic process, that glaucomatous damage to the optic disc is not always a consequence of the condition, and that the prognosis is good with modern laser and surgical approaches to management.

## 2. Why Is Malignant Glaucoma So Rare?

Classical malignant glaucoma is reported to occur in 0.4–6% cases of incisional surgery for primary angle-closure glaucoma [5–7]. Most case series of malignant glaucoma report this as a consequence of surgery in White patients with primary angle closure or primary angle-closure glaucoma. Since primary angle-closure glaucoma is a particularly common form of glaucoma in South-East Asia and China, it is remarkable that malignant glaucoma is not described as a complication of surgery in these patients with primary angle-closure glaucoma [8–11]. This may reflect a different aetiology and physiologic basis of angle closure in White and Asian subjects; a subject that has not yet been resolved [12].

Where malignant glaucoma occurs in nonsurgical situations or spontaneously, the literature contains isolated case reports and the absence of large case series of such occurrences underlines the rarity of this condition.

## 3. What Are the Clinical Features of Malignant Glaucoma?

Malignant glaucoma is diagnosed when there is shallowing of the central (axial) anterior chamber in association with increased intraocular pressure (IOP) and normal posterior segment anatomy. Absence of pupillary block needs to be confirmed by the presence of a patent iridotomy and posterior segment pathology (particularly suprachoroidal haemorrhage) should be excluded through careful fundus evaluation.

The first symptom is often an improvement in near vision secondary to a myopic shift in refraction as the lens-iris diaphragm moves forward. Malignant glaucoma can be difficult to detect early in its course before elevation in IOP occurs. In most eyes the IOP is typically greater than 21 mmHg, but in some eyes the IOP may be normal or even low [13]. Pain and inflammation occurs when the intraocular pressure rises spontaneously and corneal oedema may develop. There is an absence of forward bowing of the iris.

Ultrasound biomicroscopy (UBM) of eyes during an episode of malignant glaucoma shows anterior rotation of the ciliary processes, which press against the lens equator (or the anterior hyaloid in aphakia) and prevent forward flow of aqueous (hence the term ciliary block glaucoma) [13–15]. It is possible that malignant glaucoma is part of a spectrum of disorders associated with a small idiopathic supraciliary effusion that is partially responsible for anterior ciliary body rotation, aqueous misdirection, and anterior segment structure displacement [14]. UBM also shows that the size of the lens in some eyes with malignant glaucoma is smaller than normal, and this may allow the lens to move forward within the eye [16].

## 4. How Can Malignant Glaucoma Be Classified?

Malignant glaucoma may be classified as classic malignant glaucoma, nonphakic glaucoma, and other malignant glaucoma syndromes [17, 18].

- Classic malignant glaucoma is a rare complication of incisional surgery for primary angle-closure glaucoma [5, 6]. It can occur in phakic, aphakic, and pseudophakic eyes, and its occurrence appears to be independent of the type of surgery and the preoperative intraocular pressure level prior to surgery. The timing is variable, ranging from the immediate postoperative period to many years after surgery and may coincide with the cessation of cycloplegic drugs [19–21]. Partial or total closure of the drainage angle at the time of surgery and axial hypermetropia is associated with an increased risk [5, 22].
- Nonphakic malignant glaucoma develops in patients after cataract extraction. This group of patients includes those in whom there is persistent malignant glaucoma despite cataract extraction. It can occur in eyes with or without glaucoma [5, 23].
- Other malignant glaucoma syndromes; although usually associated with filtration surgery, malignant glaucoma may occur spontaneously [24, 25] and has also been described in a number of glaucoma-related situations. These include laser treatment (peripheral laser iridotomy [26, 27], trabeculectomy scleral flap suture lysis [28, 29], and cyclophotocoagulation [30]); use of miotics [31, 32] and trabeculectomy bleb needling [33]. There are sporadic reports of malignant glaucoma in association with infection [34, 35], retinopathy of prematurity [36, 37], retinal detachment [38], retinal vein occlusion [39], and trauma [40]. It is plausible that malignant glaucoma may arise in some of these situations following ciliary body swelling and the formation of an inflammatory barrier in the zonular-capsular region which impedes anterior flow of aqueous humour [41]. However, some isolated reports may simply represent misdiagnosis, as the mechanism of precipitating the episode of malignant glaucoma in these circumstances is unclear.

## 5. What Is the Pathogenic Mechanism Underlying Malignant Glaucoma?

Malignant glaucoma is a multifactorial condition that is thought to occur in anatomically predisposed eyes. Although poorly understood, the pathophysiology is thought to involve an alteration in the anatomic relationship of the lens, ciliary body, anterior hyaloid face, and vitreous, which results in forward movement of the iris-lens diaphragm. While the exact mechanism remains unclear, three pathogenic mechanisms have been proposed.

- Shaffer and Hoskins suggested that posterior diversion of aqueous flow causes accumulation of aqueous behind a posterior vitreous detachment with secondary forward movement of the iris-lens diaphragm [42]. He and Hoskins observed collections of fluid behind the vitreous gel, which also seemed more dense than normal, and believed that this prevented forward flow of aqueous [2]. Shaffer and Hoskins postulated a valve-like mechanism by which aqueous humour was “misdirected” posteriorly. The mechanism causing the posterior diversion of aqueous and the nature of the unidirectional valve remain unclear.
- Chandler proposed that laxity of lens zonules coupled with pressure from the vitreous leads to forward lens movement. A vicious circle is set up in that the higher the pressure in the posterior segment, the more firmly the lens is held forward [6].
- Quigley et al. proposed that the precipitating event which increases vitreous pressure is choroidal expansion [43, 44] and that the initial compensatory outflow of aqueous along the posteroanterior pressure gradient causes shallowing of the anterior chamber. Choroidal expansion has been detected on UBM in eyes with malignant glaucoma, and choroidal effusion secondary to angio-oedema has also been reported to result in malignant glaucoma [45, 46].

Whatever the mechanism, the final common pathway is the establishment of a vicious cycle whereby the transvitreal pressure cannot be equalised by outflow of aqueous humour. As the pressure rises, the anterior vitreous gel becomes less permeable to the forward movement of gel and this exacerbates the problem [47]. Fluid buildup behind the vitreous leads to vitreous condensation which exerts a forward force, resulting in anterior displacement of the lens-iris diaphragm. In small eyes prone to angle closure the forward movement would look like acute pupillary block. However, iridotomy does not reverse the situation. The concept that the lens subsequently pushes the peripheral iris into the anterior chamber angle led to the proposed term of “direct lens block glaucoma” [6].

## 6. What Are the Principles of Management of Malignant Glaucoma?

**Figure 1:**
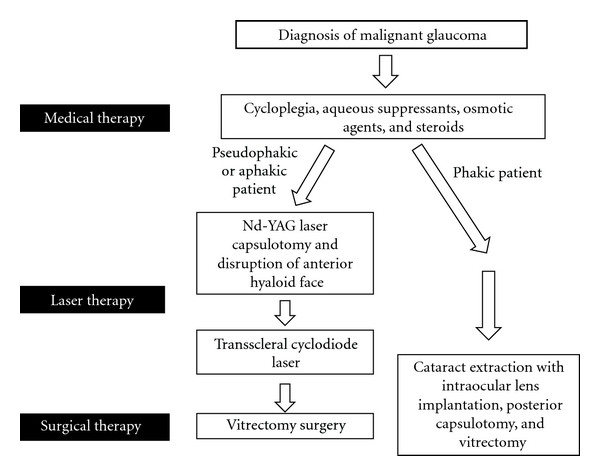
Management pathway for malignant glaucoma.

The first step is to make an accurate diagnosis and exclude the differential diagnoses of pupillary block glaucoma, a suprachoroidal haemorrhage or choroidal effusion and secondary causes of angle closure. Once the diagnosis is made, the following pathway of management can be followed (Figure 1). 

### 6.1. Medical Therapy

#### 6.1.1. Cycloplegia

Mydriatics (atropine and phenylephrine) should be given immediately in order to tighten the lens zonules and pull the anteriorly displaced lens backwards [5, 40]. This condition does not respond to the use of miotics (unlike pupillary block), which are contraindicated as they may exacerbate the situation by promoting zonular relaxation and encourage forward lens movement.

#### 6.1.2. Intraocular Pressure Reduction

 Oral acetazolamide and topical beta-blockers and alpha agonists are used to reduce aqueous production.

#### 6.1.3. Reduction of Vitreous Volume

 Osmotic agents (mannitol or glycerol) are used to reduce vitreous volume, deepen the anterior chamber, and possibly increase vitreous permeability [48].

#### 6.1.4. Anti-Inflammatory Medication

 Topical steroids can help to reduce inflammation [49]. Medical management is reported to be curative in 50% patients within a 5-day period [5]. Once the anterior chamber deepens and the IOP normalises, medical treatment can be slowly withdrawn, with the osmotic agent stopped first, then the aqueous suppressants and finally the cycloplegic medication. Indefinite cycloplegia with topical atropine or other cycloplegics may be required to prevent recurrence [50].

### 6.2. Laser Therapy

Successful laser treatment aims to restore a normal aqueous flow pattern by establishing a direct communication between the vitreous cavity and anterior chamber.

#### 6.2.1. Disruption of Anterior Hyaloid Face

 An intact hyaloid face is an important pathogenic factor in malignant glaucoma and in pseudophakic or aphakic patients, Nd:YAG laser capsulotomy with disruption of the anterior hyaloid face is often effective [15, 51–53]. UBM imaging shows that anterior rotation of the ciliary body and anterior chamber shallowing normalise after rupture of the anterior hyaloid face [15]. It has been suggested that a large optic lens (greater than 7 mm) or the presence of synechiae between the implant and capsule may prevent subsequent flow of aqueous forwards and in this situation laser capsulotomy can be performed in the region of the lens dialling hole (if present) to allow direct passage of aqueous into the anterior chamber [54].

#### 6.2.2. Laser of Ciliary Processes

 The successful use of transscleral cyclodiode laser photocoagulation in pseudophakic patients can help eliminate an abnormal vitreociliary relationship by posterior rotation of the ciliary processes secondary to coagulative shrinkage [55, 56]. Often a single session of therapy is sufficient over 1-2 quadrants [56]. An alternative option is direct argon laser treatment of the ciliary processes through a peripheral iridotomy [57]. Cyclocryotherapy has been used in the past but no longer has a place in modern management [58].

### 6.3. Surgical Therapy

In malignant glaucoma that is refractory to medical and laser therapy, surgical intervention to remove the vitreous is necessary to increase aqueous flow into the anterior chamber [50, 59]. The success of this was first described by Chandler, who reported a method of vitreous aspiration through an 18-guage needle via an incision through the pars plana [60]. With time, this principle of therapy has evolved into current pars plana vitrectomy surgery techniques.

The key role of removal of the anterior vitreous is demonstrated by the fact that core vitrectomy surgery leads to resolution of malignant glaucoma in only 25–50% of the phakic eyes compared to 65–90% pseudophakic eyes [13, 61, 62]. This probably reflects the lack of effective removal of the anterior hyaloid in phakic eyes because of the risk of lens damage and subsequent cataract formation. A technique of intraocular videoendoscope-guided, fluorescein-assisted pars plana vitrectomy that allows direct visualisation and thorough removal of the anterior vitreous without the need for lens extraction in prepresbyopic patients has been successfully used in malignant glaucoma [63].

Intracapsular cataract extraction has been reported to be successful in 50% cases [61]. Combined cataract extraction and vitrectomy in phakic eyes can increase the success rate from 25% to 83% if the posterior capsule is removed [62]. The key feature is that the posterior capsule must be breached and the anterior vitreous removed, so that the relationship between the vitreous and ciliary body that predisposes to the vicious cycle of malignant glaucoma is disrupted. Therefore, in phakic patients, definitive management requires phacoemulsification surgery plus intraocular lens implantation combined with removal of the posterior capsule at time of vitrectomy [49, 60, 61]. A staged surgical approach may help overcome technical difficulties with phacoemulsification surgery in the presence of a shallow anterior chamber and high intraocular pressure. A preliminary core vitrectomy is initially done to debulk the vitreous, soften the eye, and deepen the anterior chamber, followed by standard phacoemulsification surgery and intraocular lens implantation. Finally, residual vitrectomy and hyaloidectomy with removal of the retrolental posterior capsule are performed [64]. Some authors advocate the additional step of zonulohyaloidectomy [65]. In resistant cases, pars plana glaucoma tube insertion has been described [66].

Since malignant glaucoma often follows trabeculectomy surgery, there is a risk of long-term failure of the filtration bleb as a consequence of any additional surgical procedures needed to treat malignant glaucoma. Byrnes et al. recorded bleb failure in 16% eyes following pars plana vitrectomy [61]. Careful followup is needed to monitor this, so that appropriate intervention can be instituted if needed.

### 6.4. Management of the Fellow Eye

After an episode of malignant glaucoma in one eye, there is a high risk of this complication occurring in the fellow eye after a surgical intervention [67]. The patient should be warned of this when consent is taken for surgery on the fellow eye. Prophylactic measures include cessation of miotic drops (which cause ciliary body swelling and anterior rotation of the lens-iris diaphragm), prolonged use of atropine after trabeculectomy surgery (with careful monitoring after this is stopped), and avoidance of anterior chamber shallowing in the postoperative period (using anterior chamber viscoelastic and tight scleral flap suturing). Preoperatively, a prophylactic laser peripheral iridotomy is recommended by some (although this can itself occasionally lead to malignant glaucoma) [23]. These methods will help maintain the normal anatomical position of the iris-lens diaphragm and reduce the risk of aqueous misdirection.

In eyes with primary angle-closure glaucoma that is not medically controlled, lens extraction may be undertaken as a primary procedure, rather than filtration surgery [9]. The former has a lower risk of malignant glaucoma and, if it occurs, the management is easier in a pseudophakic eye. Some specialists have advocated prophylactic pars plana vitrectomy at the time of phacoemulsification with intraocular lens implantation to prevent malignant glaucoma in high-risk fellow eyes [68].

## 7. Conclusion

Malignant glaucoma continues to provide a therapeutic challenge, which probably reflects our limited understanding of its aetiology. It is curious that this condition appears to be extremely rare in Asians, where the prevalence of primary angle-closure glaucoma is common. Whatever the true mechanism, the fact that it is relieved when a direct communication is made between the anterior chamber and vitreous cavity supports the theory that the lens, anterior vitreous, and ciliary processes are intimately involved in the pathogenesis. The fact that it often occurs in fellow eyes suggests that it is a tendency in an individual rather than a random event. All eyes with primary angle-closure glaucoma should be closely followed, particularly in the early postoperative period following glaucoma drainage surgery. If a patient develops malignant glaucoma in one eye, preventive measures should be taken to prevent it occurring in the fellow eye at the time of surgery. The prognosis of this condition is good with currently available treatment modalities and malignant glaucoma no longer deserves its historical name.
